# Bloodstream infections caused by multidrug-resistant non-fermentative bacilli in southern of Poland in 2013

**DOI:** 10.1186/2047-2994-4-S1-P138

**Published:** 2015-06-16

**Authors:** A Chmielarczyk, M Pomorska-Wesołowska, M Pobiega, G Ziółkowski, D Romaniszyn, J Wojkowska-Mach

**Affiliations:** 1 Jagiellonian University Medical School, Kraków, Poland; 2 Department of Microbiology, Analytical and Microbiological Laboratory, Korlab Nzoz, Ruda Slaska, Poland; 3 Higher School of Medicine in Sosnowiec, Sosnowiec, Poland

## Introduction

Modern medicine requires a clear and explicit criteria to describe the phenomena of public health; and one of the major problems of public health is drug resistance of microorganisms.

## Objectives

The aim of this study was to analyze how big epidemiological problem are highly-resistant – multidrug-resistant (MDR) and extensively-drug resistant (XDR) non-fermentative bacilli isolated from bloodstream infections (BSI) in southern Poland.

## Methods

The study comprised consecutive, non-repetitive non-fermentative bacilli (NFB) isolates received by the Chair of Microbiology UJCM with collaborative 2 laboratories in 2013 from BSI of hospitalized (12 hospitals) throughout south of Poland (Malopolska and Silesia).

Studied strains from BSI were belonging to groups: ACI (*Acinetobacter baumannii* n=21, *Acinetobacter lwoffii* n=1, *Acinetobacter ursingi* n=1), PAR (*Pseudomonas aeruginosa* n=12), and others (*Stenotrophomonas malthophilia* n=10, *Achromobacter denitrificans* n=5, *Comamonas testosterone* n=1, *Ochrobactrum anthropi* n=1).

Antimicrobial susceptibility was assessed according to current EUCAST guidelines. Different patterns of resistance were defined according to Magiorakos (2012) as: MDR strains or XDR.

## Results

NFB strains occurred with different frequencies, the highest prevalence was associated with ACI: 1.3%.

More than 75% of ACI strains were resistant to 14 out of 16 antimicrobials, among ACI also found the highest share of XDR: 95.7%. The most of these isolates were resistant to all antibiotics with the exception of colistin: MIC50 for colistin was 1.

In the group of PAR isolates 91.7% were XDR, MIC50 for colistin was 1.

DiversiLab typing demonstrated the presence of two dominant ACI clones. Clone 1 and clone 2 , both are classified as the European clone 2 (EUII).

## Conclusion

The presented data indicate a high potential therapeutic problems related to the large resistance of ACI isolates. The used stratification of drug resistance (MDRO/XDR/PDR) may become an important tool for the assessment of public health and microbiological hazards at different levels. (supported by DEC-2012/05/B/NZ7/02880).

## Disclosure of interest

None declared.

